# Editorial: Metabolic and Neurological Modulation by Glucosinolates, Isothiocyanates, and Terpenes to Improve Health

**DOI:** 10.3389/fphar.2022.915223

**Published:** 2022-05-03

**Authors:** M. E. González-Trujano, M. Déciga-Campos, J.C. Tavares-Carvalho, D. A. Moreno

**Affiliations:** ^1^ Laboratorio de Neurofarmacología de Productos Naturales, Dirección de Investigaciones en Neurociencias del Instituto Nacional de Psiquiatría “Ramón de la Fuente Muñiz”, Ciudad de México, Mexico; ^2^ Sección de Estudios de Posgrado e Investigación, Escuela Superior de Medicina, Instituto Politécnico Nacional, Ciudad de México, Mexico; ^3^ Laboratorio de Pesquisa em Fármacos, Curso de Farmacia, Departamento de Ciencias Biológicas e da Saude, Universidade Federal do Amapa, Macapa, Brazil; ^4^ Laboratorio de Fitoquímica y alimentos saludables (Lab FAS), Departamento de Ciencia y Tecnología de Alimentos, CEBAS-CSIC, Murcia, Spain

**Keywords:** antioxidants, food and natural products, inflammation, nanoformulation, non-communicable and neurological diseases, transcriptonal signals

Foods and natural products from plant origin for developing ingredients and formulations for prevention and treatment of diseases is an area of growing interest, especially in the management of chronic noncommunicable and neurological diseases. These conditions need of long-term interventions and result of a combination of genetic, physiological, environmental, and behavioral factors.

In a complete food or a plant extract or a fraction, or even an isolated bioactive compound, after structure elucidation, the investigation for better efficacious and safe therapeutic uses is needed. The example of the glucosinolates, are secondary plant metabolites abundantly found in plant order Brassicales, and precursors of chemopreventive isothiocyanates, has demonstrated to reduce oxidative stress and inflammation *via* Keap1-Nrf2-ARE-mediated induction of phase 2 cytoprotective enzymes, but little is known regarding the association between glucosinolate intake and risk of non-communicable diseases (Type 2 diabetes, obesity, and other metabolic syndromes associated with lipid and glucose metabolism), and neurological diseases (Connolly et al.). Most natural glucosinolates resident in plants, with more than 130 different glucosinolates already validated. Terpenes are natural products with cannabinoid and opioid mode of action opening the possibility for their application in treating metabolic syndromes and neurological diseases (Philippot et al.; Del prado-Audelo et al.).

This Research Topic aimed to integrate new knowledge on the association between the intake of glucosinolates or diterpenes such as neoclerodanes and the incidence of non-communicable and neurological diseases from the different aspects involved in their functionality: bioaccesibility, bioavailability, metabolism, and functionality (biological activity), especially in modes of action on specific targets of interest for future therapies and dietary interventions.

An increasing body of evidence highlights the strong potential for a diet rich in fruit and vegetables to delay, and often prevent, the onset of chronic diseases, including cardiometabolic, neurological, and musculoskeletal conditions, and certain cancers. Glucosinolates and isothiocyanates act *via* several mechanisms, ultimately exhibiting anti-inflammatory, antioxidant, and chemo-protective effects. The incorporation of glucosinolate-rich foods to daily diet is linked to a reduced incidence of chronic diseases, but future large-scale placebo-controlled human trials including standardized glucosinolate supplements are needed (Connolly et al.).

In the last decades, the search for natural products with biological applications as alternative treatments for several inflammatory diseases has increased. In this respect, terpenes are a family of organic compounds obtained mainly from plants and trees, such as tea, cannabis, thyme, and citrus fruits like lemon or mandarin. These molecules present attractive biological properties such as analgesic and anticonvulsant activities. Furthermore, several studies have demonstrated that certain terpenes could reduce inflammation symptoms by decreasing the release of pro-inflammatory cytokines, for example, the nuclear transcription factor-kappa B, interleukin 1, and the tumor necrosis factor-alpha. Thus, due to various anti-inflammatory drugs provoke side effects, the search and analysis of novel therapeutics treatments are attractive. An analysis of terpenes’ chemical structure and their mechanisms in anti-inflammatory functions are addressed in (Del prado-Audelo et al.), where a very recent analysis of investigations on their applications as alternative treatment agents for inflammatory diseases is well described (Nguyen et al., 2020). Furthermore, in this on the possibility of using nanoformulations offers a state-of-the-art opportunity for future industrial applications, after a deeper evaluation of safety of the new developed therapeutics.

Δ9-tetrahydrocannabinol (THC) is the main psychoactive constituent of cannabis, one of the most used drugs during pregnancy and lactation that efficiently crosses the placental and blood-brain barriers. Despite the recent legalization initiatives worldwide, the adverse outcome pathway (AOP) of THC following exposure during brain development, is incompletely understood. A single injection of THC on postnatal day (PND) 10 altered adult spontaneous behavior and habituation rates in adult mice. Similar behavioral alterations have been reported following PND 10 exposures to the commonly used over-the-counter analgesic acetaminophen (AAP; also known as paracetamol); as both THC and AAP interact with the endocannabinoid system (Philippot et al.) reported e that a single THC dose on PND 10 decreased transcript levels of Trkb 24 h after exposure in both the frontal and parietal cortex, and in the hippocampus in mice. Effects on the Nrf2-Keap1 axis were also found in both the parietal cortex and hippocampus following neonatal exposure to THC. THC exposure also increased transcript levels of Cb1r in the parietal cortex and increased pro apoptotic protein BAX in the frontal cortex. Authors also emphasized the importance of this study because of mainly three reasons: 1) information from the developmental neurotoxic AOP of THC where transcriptional changes of the neurotrophic receptor Trkb are central, 2) the PND 10 exposure model provides information relevant to the exposures happening in humans and 3) since PND 10 exposure to AAP also decreased Trkb transcript levels, it suggests THC and AAP may share key events in their respective AOP through endocannabinoid-mediated alterations of the BDNF-TRKN signaling pathway.

Depression is a widespread chronic medical illness affecting thoughts, mood, and physical health. However, the limited and delayed therapeutic efficacy of monoaminergic drugs has led to intensive research efforts to develop novel antidepressants. In the work of (Liu et al.), a multidisciplinary approach was used to explore the antidepressant-like actions of ARN-3236 in mice. Chronic social defeat stress (CSDS) and chronic unpredictable mild stress (CUMS) models of depression, various behavioral tests, high performance liquid chromatography-tandem mass spectrometry, stereotactic infusion, viral-mediated gene transfer, western blotting, co-immunoprecipitation, and immunofluorescence were used together. It was found that ARN-3236 could penetrate the blood-brain barrier. Repeated ARN-3236 administration induced significant antidepressant-like effects in both the CSDS and CUMS models of depression, accompanied with fully preventing the stress-enhanced SIK2 expression and cytoplasmic translocation of cyclic adenosine monophosphate response element binding protein (CREB)-regulated transcription coactivator 1 (CRTC1) in the hippocampus. ARN-3236 treatment also completely reversed the down-regulating effects of CSDS and CUMS on the hippocampal brain-derived neurotrophic factor (BDNF) system and neurogenesis. Also concluded by the authors of this study, the ARN-3236 possesses strong protecting effects against chronic stress and could be a novel antidepressant beyond monoaminergic drugs.

Lastly, but of very relevant interest, the acute ischemic stroke is a serious disease that endangers human health. The work of (Zhang et al.) described that TPPU (trifluoromethoxyphenyl-3-(1-propionylpiperidin-4-yl) urea (TPPU), protects the brain against focal ischemia in rats suggesting it might be an effective therapy in humans. In this investigation, authors explored the TPPU mechanism of action by assessing whether it could preserve blood-brain barrier integrity and reduce apoptosis in the brain during permanent middle cerebral artery occlusion in male Sprague-Dawley rats. TPPU administration at the onset of stroke and once daily thereafter led to smaller infarct volume and brain edema as well as milder neurological deficits. TPPU significantly inhibited the activity of soluble epoxide hydrolase and matrix metalloproteases 2 and 9, reducing 14,15-DHET levels, while increasing expression of tight junction proteins. TPPU decreased numbers of apoptotic cells by down-regulating the pro-apoptotic proteins BAX and Caspase-3, while up-regulating the anti-apoptotic protein BCL-2. These results suggest that TPPU can protect the blood-brain barrier and reduce the apoptosis of brain tissue caused by ischemia.

New scientific evidence still remains to be documented on how glucosinolates can benefit human health by consuming vegetables and condiments of the Brassicaceae family. Toxicity and safety studies are also required to guarantee therapeutic potential at the preclinical and clinical levels, respectively. In addition, clinical research that allows documenting the impact that vegetables have on nutrition and as a functional food to establish the appropriate concentrations to reduce oxidative or degenerative effects are also important. As well as the studies that relate the therapeutic efficacy and their pharmacokinetic parameters to document the required amounts of the bioactive compounds that must be ingested to impact on the non-communicable and neurological diseases.

In this Research Topic, it was not a purpose to document purification and characterization of novel and potential bioactive glucosinolates, isothiocyanates, or terpenes. However, it will be interesting to document this information. In particular, novel analytical methods of these constituents migh be relevant since the known methods are expensive, slow, and laborious. Therefore, it is interesting to investigate other simple analytical techniques that allow the extraction of these compounds that are of therapeutic and agricultural interest. Additionally, metabolomic studies with genome-bases functional characterization of these plant natural products to modern pharmaceutical technology are other relevant topic to be described.

A better understanding of the biological properties of these compounds, their pharmacokinetics, pharmacodynamics, and chemoprotection in humans, will allow more rational and specific prescriptive and personalized nutrition: “green chemoprotection” or “frugal medicine".
